# RACIAL-ETHNIC DISPARITIES IN PHYSICAL PERFORMANCE: THE STUDY OF WOMEN’S HEALTH ACROSS THE NATION

**DOI:** 10.1093/geroni/igz038.1987

**Published:** 2019-11-08

**Authors:** Carrie Karvonen-Gutierrez, Alicia Colvin, Andrea Stewart, Bradley Appelhans, Jane Cauley, Sheila Dugan, Samar El Khoudary, Barbara Sternfeld

**Affiliations:** 1 University of Michigan, Ann Arbor, Michigan, United States; 2 Department of Epidemiology, Graduate School of Public Health, University of Pittsburgh, Pittsburgh, Pennsylvania, United States; 3 Rush University Medical Center, Chicago, Illinois, United States; 4 Department of Epidemiology, Graduate School of Public Health, Pittsburgh, Pennsylvania, United States; 5 Division of Research, Kaiser Permanente, Kensington, California, United States

## Abstract

Racial/ethnic disparities exist in physical performance among older adults, but little is known about the factors leading to these differences. We evaluated how sociodemographic, health, behavioral, and psychosocial factors mediated the relationship between race/ethnicity and physical functioning performance among 1,855 Black, Chinese, Hispanic, Japanese and White postmenopausal women from the Study of Women’s Health Across the Nation. White women had better mean physical performance scores (decile-based score including relative performance on grip strength, timed 4-meter walk, and timed repeat chair stand tasks) as compared to Black, Hispanic and Chinese women but slightly poorer scores vs. Japanese women. In Blacks and Hispanics, 75% and 95% of that disparity, respectively, was through mediators, particularly education, financial strain, BMI, physical activity and pain. Addressing issues of poverty, racial inequality, bodily pain and obesity could reduce some racial/ethnic disparity in functional limitations as women age.

